# Dependency in Activities of Daily Living During the First Year After Stroke

**DOI:** 10.3389/fneur.2021.736684

**Published:** 2021-11-08

**Authors:** Hannah E. Wurzinger, Tamar Abzhandadze, Lena Rafsten, Katharina S. Sunnerhagen

**Affiliations:** ^1^Department of Clinical Neuroscience, Institute of Neuroscience and Physiology, Sahlgrenska Academy, University of Gothenburg, Gothenburg, Sweden; ^2^Department of Occupational Therapy and Physiotherapy, Sahlgrenska University Hospital, Gothenburg, Sweden; ^3^Centre for Person-Centred Care (GPCC), University of Gothenburg, Gothenburg, Sweden

**Keywords:** activities of daily living, longitudinal studies, outcome assessment, stroke rehabilitation adherence, prognosis, p-ADL, cross validation (CV), logistic regression

## Abstract

**Background:** Dependency in personal activities of daily living (ADL) is a common short-term and long-term consequence of stroke and requires targeted rehabilitation. As the duration of hospital stay has become shorter in recent decades, early identification of patients who require rehabilitation has become vital. To our knowledge, no study has investigated whether ADL dependency in the very early stages after admission to the stroke unit can explain ADL dependency 3 and 12 months later. This knowledge would facilitate planning for very early discharge and patient-centered rehabilitation.

**Objective:** This study evaluated whether ADL dependency within 2 days after stroke could explain ADL dependency at 3 and 12 months after stroke.

**Methods:** This longitudinal cohort study included patients with stroke who were treated at a stroke unit in the Sahlgrenska University Hospital (Gothenburg, Sweden) between May 2011 and March 2016. The primary independent variable was ADL dependency at 36–48 h after admission to the stroke unit, which was assessed using a Barthel Index (BI) score of ≤90. The dependent variables were self-reported personal ADL dependency at 3 and 12 months after stroke. Binary logistic regression analyses were performed.

**Results:** Of 366 eligible patients (58% male; median age 71 years), a majority (76%) had mild stroke and 60% were ADL dependent 36–48 h after stroke. Univariable and multivariable logistic regression analyses showed that patients who were dependent within the first 2 days after stroke had higher odds for being dependent 3 months as well as 12 months after stroke.

**Conclusion:** The results indicated that dependency in personal ADL during the first 2 days can explain dependency at 3- and 12-month post-stroke. Therefore, early ADL assessments post-stroke can be used for understanding rehabilitation needs after stroke.

## Introduction

Cerebrovascular diseases are amongst the most prevalent causes of disability (1). Although the age-adjusted rates of stroke are decreasing, the total number of strokes are increasing due to global population growth and aging populations (2, 3). Furthermore, the early stroke fatality rate is decreasing (4), leading to an increasing number of stroke survivors with stroke-related disability and years lived with disability (5). Early rehabilitation is the key in reducing the burden of stroke-related disability.

Activities of daily living refer to various tasks and activities that people perform on daily basis (6). They can be grouped into instrumental activities of daily living (e.g., shopping, paying the bills) and personal activities of daily living (referred to as ADL in this article e.g., eating, getting dressed). ADL dependency is a common consequence post-stroke and persists in 35% of stroke survivors during the first year after stroke (7). Increased disability after stroke has been linked to various factors, including older age (8), co-morbidity (8), impaired cognition (9), and stroke severity at onset (10). However, patients with mild stroke can also experience ADL dependency in everyday life (11) and have unmet rehabilitation needs (12). Hence, prognosis cannot be accurately estimated based on stroke severity alone.

Most of the recovery in ADL typically occurs within the first 6 weeks after stroke and is related to initial stroke severity (13, 14). In the later stages of stroke, there is generally no decline or improvement in ADL (15, 16). Studies have shown that patients with ADL dependency during the first week after stroke are also dependent at 6 months and 3 years after stroke (8, 17). However, the explanatory value of ADL assessment very early after stroke has not been thoroughly investigated. This is important as the length of stay in Swedish hospitals has decreased in recent decades (18). The current median length of hospital stay is 7 days (19). Patients with mild-to-moderate stroke can be discharged shortly after admission, resulting in a limited period for the assessment of rehabilitation needs and prognosis.

While very early ADL assessments are routinely performed in stroke units, it is unclear how the results of these assessments are related to long-term outcomes. The finding of a positive association between very early ADL assessments and ADL ability in the later stages of stroke would facilitate planning for patient-centered rehabilitation and early hospital discharge. Therefore, the present study evaluated whether ADL assessments within 2 days after admission to the stroke unit could explain dependency in personal ADL 3 and 12 months after stroke.

## Materials and Methods

### Study Design

This longitudinal cohort study evaluated data from a research database of patients who were treated at one stroke unit in the Sahlgrenska University Hospital (SU) between May 2011 and March 2016 (20, 21). The SU is the largest hospital in western Sweden. It is a regional center for neurosurgery and thrombectomy. The research database was linked to the Swedish national stroke quality register, Riksstroke (22). Data pertaining to acute care parameters and self-reported outcomes at 3 and 12 months after stroke were collected. A statistician affiliated with the Riksstroke registry performed data linkage by using each patient's unique personal identification number. The data used for the present study did not contain personal identification numbers or other identifiable information.

The inclusion criteria comprised: confirmed stroke diagnosis according to the World Health Organization criteria; age ≥ 18 years; availability of ADL assessment results obtained within 36–48 h after admission to the stroke unit; and availability of the completed Riksstroke acute form. Data from patients who passed away during their hospital stay were not analyzed.

### Ethics

The study complied with the Declaration of Helsinki and was approved by the Gothenburg Regional Ethical Review Board (http://www.epn.se/sv/goeteborg/om-naemnden/, reference number: 042–11, amendment: T966-17). The Swedish Data Protection Authority does not require informed consent for research use of registry data. In addition, the Personal Data Act (Swedish law #1998:204, issued April 29, 1998) allows medical chart data to be collected for clinical research and quality control purposes without written informed consent.

### Data Availability Statement

According to the Swedish regulations (https://etikprovning.se/for-forskare/ansvar/), complete data cannot be made publicly available for ethical and legal reasons. However, researchers can submit requests for data to the principal investigator (contact: ks.sunnerhagen@neuro.gu.se).

### Data Collection

Patients were screened for ADL dependency and cognitive impairment by occupational therapists working at the stroke unit at SU, within 36–48 h after admission. Data pertaining to the patients' initial neurological status (as assessed by physicians) and ischemic stroke classification were extracted from patients' medical charts. The Riksstroke acute form, which was completed by research nurses working at the stroke unit, was used to collect data regarding medical treatments (thrombolysis and thrombectomy), comorbidities, living conditions, and ADL dependency before stroke. Self-reported outcomes at 3 and 12 months were collected from the Riksstroke self-administered questionnaires that were sent to the patients.

### Study Variables

The dependent variables comprised ability to perform personal ADL at 3 and 12 months after stroke. This was evaluated using self-administered patient questionnaires. Dependency in personal ADL was considered present based on a response indicating dependency in one or more of the following activities: mobility, using the toilet, and getting dressed or undressed.

The primary independent variable was ADL dependency, which was assessed using the Barthel Index (BI) (23) at 36–48 h after stroke. The BI score ranges from 0 to 100, with a higher score indicating a higher level of ADL independence. ADL dependency is defined by scores of ≤90 (24).

Neurological symptoms were evaluated at admission to SU. The National Institutes of Health Stroke Scale (NIHSS) was used. NIHSS is a stroke scale with scores ranging from 0 to 42, where 0 indicates no neurological deficits (25). Mild stroke is identified based on scores of ≤3 (26). The Montreal Cognitive Assessment (MoCA) was performed within 36–48 h after admission to the stroke unit. The MoCA is a cognitive screening instrument with scores ranging from 0 to 30; a score of 30 indicates the absence of cognitive impairment (27), while a score of ≤25 indicates the presence of cognitive impairment (27). The following data were also collected: age at stroke onset, smoking status, diabetes, hypertensive treatment, accommodations and ADL dependency before the stroke, discharge destination following discharge from the stroke unit, length of hospital stay, and ischemic stroke classification according to the Oxfordshire (Bamford) Community Stroke Project classification system (28).

### Statistics

The Mann–Whitney *U*-test for ordinal variables and Pearson's chi-squared test for nominal variables were used for drop-out analyses (baseline vs. 3 months follow-up and baseline vs. 12 months follow-up) and for comparing dependent and independent patients 36–48 h after admission to the stroke unit. McNemar's test was used to compare the following four dichotomized variables: ADL dependency before the stroke and 36–48 h, 3 months, and 12 months after the stroke.

**Figure 1:**
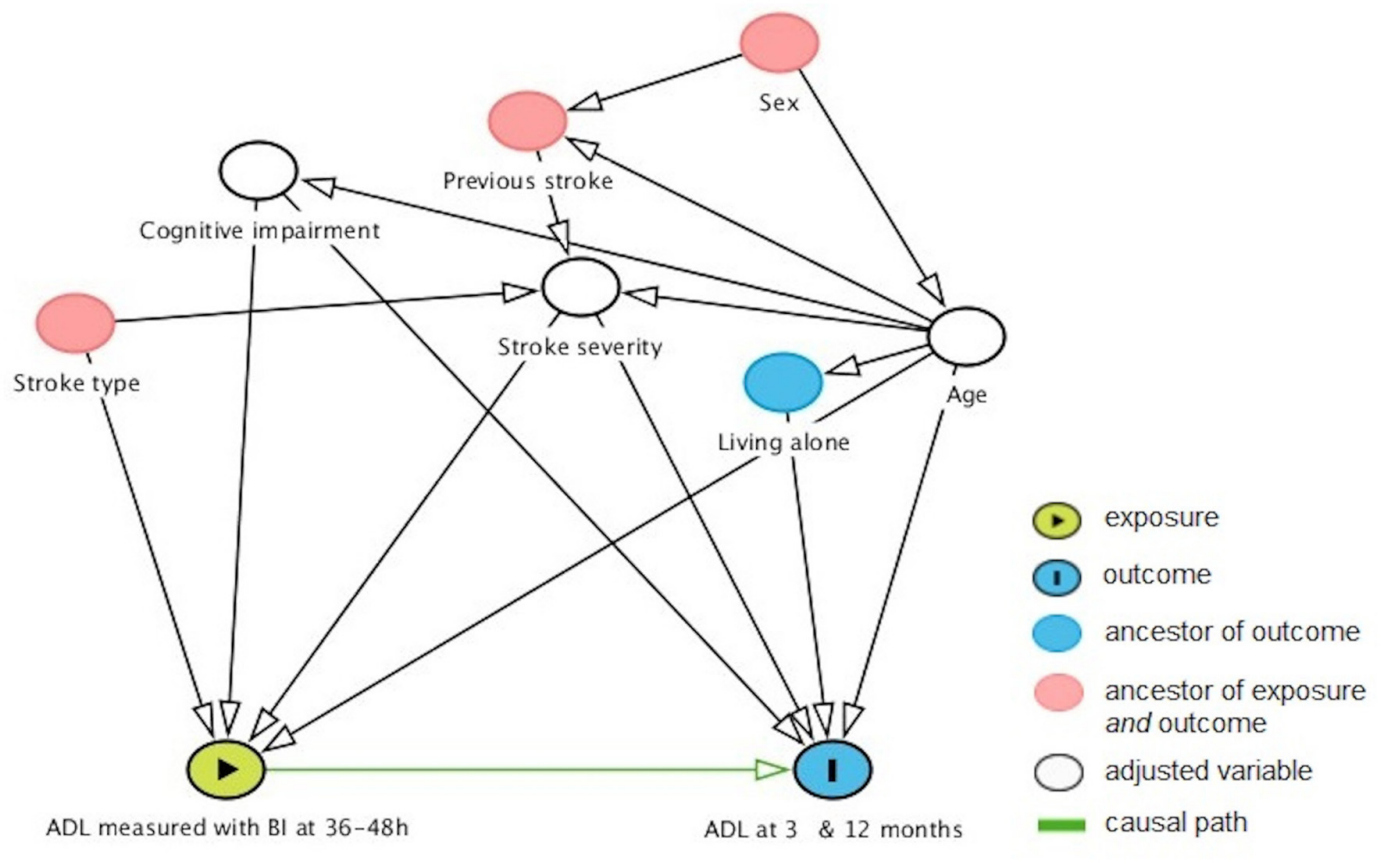
Directed acyclic graph showing factors that might confound the relationship between early and later ADL outcomes. Age, stroke severity, and cognitive impairment were identified as the minimal sufficient adjustment set. ADL, activities of daily living; BI, Barthel Index.

Binary logistic regression analyses were performed to explain ADL dependency 3 and 12 months (coded as one) after stroke. The independent variables were selected based on previous studies (9, 29) and a discussion between the authors, who have a broad range of experience with stroke rehabilitation and research. The primary explanatory variable was baseline ADL dependency. A directed acyclic graph was used to select secondary explanatory variables (age, stroke severity, and cognitive impairment) for the analysis (Figure 1).

The assumptions of the binary logistic regression were assessed by exploring multicollinearity between independent variables with Spearman's rank correlation test. Correlation coefficients between variables < ±0.7 (30) were accepted for inclusion in the regression model.

Univariable and multivariable binary regression models were built for explaining dependency at 3 and 12 months after stroke. In the univariable models, only the primary exploratory variable, ADL, assessed with BI 36–48 h after stroke, was entered. In the multivariable models, both primary and secondary explanatory variables were entered (age, NIHSS, and MoCA, full scores). The results on the variable level are reported with odds ratio (OR), 95% confidence intervals (95% CI), and *p* values.

For the full model, the analyses were performed as follows:

° The binary logistic regression model was fitted.
° The Receiver Operating Characteristic (ROC) analysis was performed with dependent variables as a state variable and predicted probabilities as a test variable. The best threshold for optimal sensitivity and specificity was identified by evaluating the coordinates of the ROC curves.
° The regression model was fit again. We used the classification threshold identified from the previous step. Sensitivity, specificity, positive predictive value (PPV), negative predictive value (NPV), and Youden's index (Yi, [sensitivity (%) + specificity (%) – 100]) of the model were reported.
° The AUC of the model was evaluated, AUC > 0.7 indicated a very good fit (30).

*Cross-validation*. The 10-fold cross-validation was performed. The dataset was divided into 10 folds. Each fold contained 90% of the data and 10% of the data was set as a holdout set. The cross-validation process was as follows:

° The model was fitted for a given 90% subset of data and identified the best threshold for balancing of Yi (sensitivity + specificity – 1).
° The threshold was further tested on the holdout set (10%). Sensitivity, specificity, positive predictive value (PPV), negative predictive value (NPV), and Yi of the model were reported.
° The AUC of the model fitted on the holdout set was evaluated, AUC > 0.7 indicated a very good fit of the model (30).
° All abovementioned steps were performed for each fold separately and aggregated results were reported. Mean and standard deviation (S.D.) were used for reporting the results, as cross-validation results cannot be assumed as independent.

Analyses were performed using IBM SPSS (software version 26.0, IBM Corp., Armonk, NY. The software license was provided by the University of Gothenburg) and R software (R Core Team, version 4.0.2, R Foundation for Statistical Computing, Vienna, Austria. R can be downloaded free of charge at https://cran.r-project.org/bin/windows/base/). The level of statistical significance was set at α = 5%.

## Results

### Study Sample

**Figure 2:**
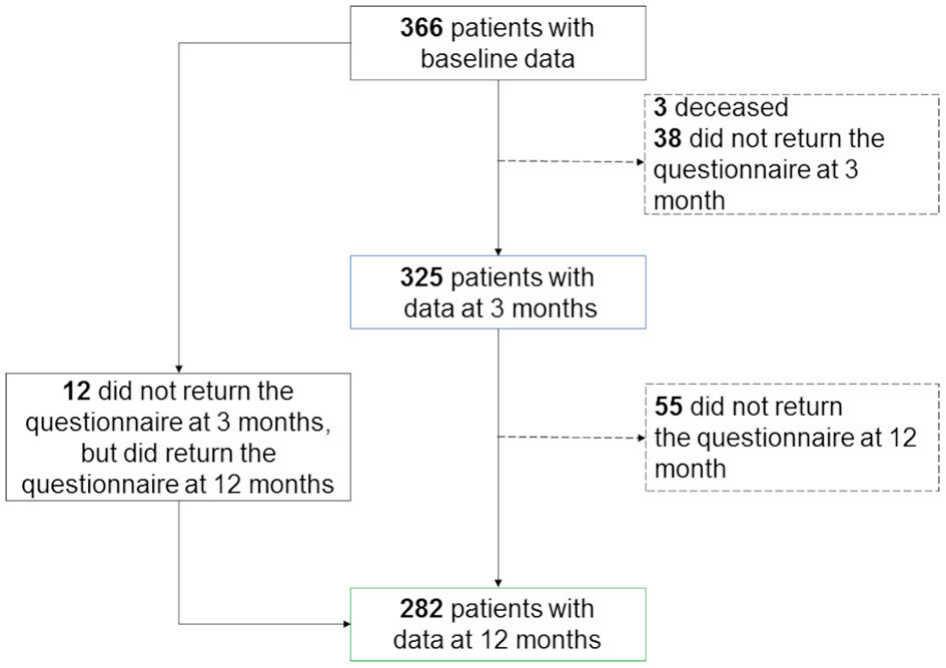
Study flowchart.

Baseline data were available for 366 patients (Figure 2), 3-month outcome data were available for 325 patients, and 12-month outcome data were available for 285 patients. There were no significant differences between the groups with and without outcome data in terms of sex, age, stroke severity, and BI scores.

The 366 patients with baseline data (42% female) had a median age of 71 years (range 19–97 years) and typically had a mild stroke (76%, NIHSS score of ≤ 3) (Table 1). Patients with early ADL dependency (BI score of ≤ 90 within 36–48 h after stroke unit admission) were more likely to be older (*p* < 0.001), have experienced more severe strokes (*p* < 0.001), and have longer hospital stays (*p* < 0.001) (Table 1).

**Table 1:** Baseline characteristics of the 366 patients.

	**Total** **(***n*** = 366)**	**Barthel index**		***p*** **-value**
		**≤90 (***n*** = 159)**	**≥95 (***n*** = 207)**	
Female sex, *n* (%)	153 (42)	75 (47)	78 (38)	0.07^a^
Age in years, mean (standard deviation)	69 (15)	75 (13)	64 (15)	**<0.001^b^**
Living situation/condition pre-stroke, *n* (%)				
Lives at home without help	334 (91)	134 (85)	200 (97)	**<0.001^a^**
Independent in activities of daily living	354 (97)	151 (95)	203 (98)	0.10
Living alone	155 (42)	84 (53)	71 (34)	**<0.001^a^**
Risk factors, *n* (%)				
Diabetes	47 (13)	22 (14)	25 (12)	0.62^a^
Smoking	55 (16)	16 (11)	39 (20)	**0.02^a^**
Previous stroke	64 (18)	32 (21)	32 (16)	0.23^a^
Hypertensive treatment	184 (51)	91 (58)	93 (46)	**0.02^a^**
Stroke type, *n* (%)				0.68^a^
Hemorrhagic	32 (9)	15 (9)	17 (8)	
Ischemic classification^c^, *n* (%)				
Total anterior circulation	3 (1)	2 (1)	1(0.5)	
Partial anterior circulation	54 (15)	29 (18)	25 (12)	
Posterior circulation syndrome	122 (33)	60 (38)	62 (30)	
Lacunar syndrome	155 (42.3)	53 (33)	102 (49)	
Treatments, yes, *n* (%)				
Revascularization (Thrombolysis or/and thrombectomy)	81 (23)	39 (25)	42 (21)	0.33^a^
Thrombolysis	72 (20)	37 (23)	35 (17)	
Thrombectomy	22 (6)	13 (8)	9 (4)	
Stroke-related outcomes, median (range[Q1–Q3])				
National Institutes of Health Stroke Scale^d^	2(0–19[0–3])	2(0–19[1–5])	1(0–14[0–2])	**<0.001^b^**
Barthel Index^e^	95(10–100[80–100])			
Montreal cognitive assessment^e^	24(3–30[20–27])	22(4–30[18–25])	25(3–30[23–27])	**<0.001^b^**
Length of hospital stay in days	7(0–43[4–11])	10(1–43[5–17])	5(0–20[4–8])	**<0.001^b^**
Discharge destination, *n* (%)				**<0.001^a^**
Own home	317 (87)	122 (77)	195 (94)	
Nursing home	15 (4)	11 (7)	4 (2)	
Another acute clinic	9 (2)	4 (3)	5 (2)	
Geriatric/rehabilitation clinic	25 (7)	22 (14)	3 (1)	

a *Pearson's chi-squared test*;

b *Mann–Whitney U test*;

c *According to the Oxfordshire Community Stroke Project classification system*;

d *Assessed at ≤ 24 h after admission*;

e *Assessed within 36–48 h after admission. Bold text indicates significant results. Data were missing for the following variables, diabetes (seven patients, 2%), smoking (20 patients, 5%), previous stroke (eight patients, 2%), hypertensive treatment (seven patients, 2%), revascularization (seven patients, 2%), and National Institutes of Health Stroke Scale score (10 patients, 3%)*.

**Figure 3:**
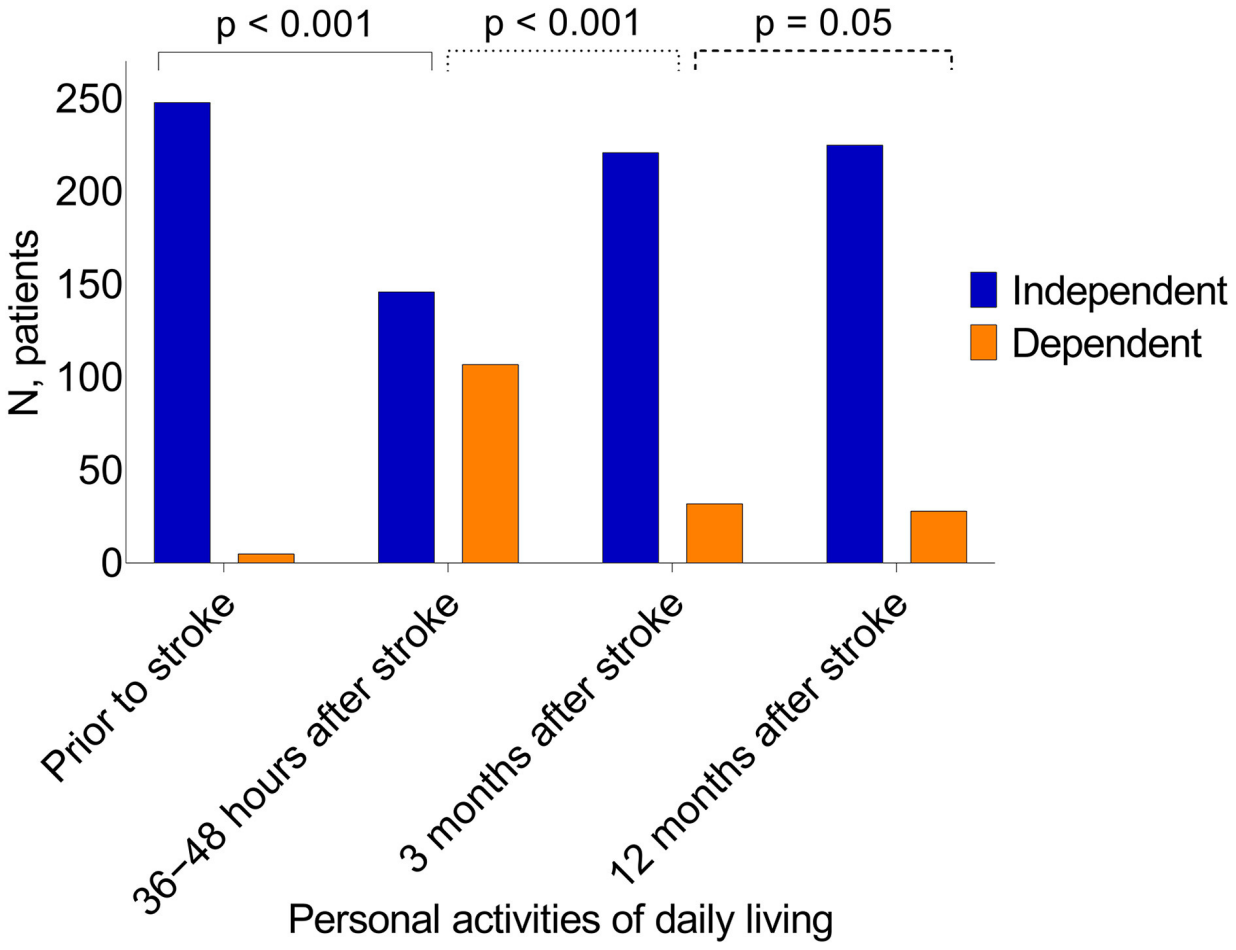
Activities of daily living performance at four different time points. Statistics, McNemar's test, *P*-values indicate statistical difference between following time points, ADL dependency before the stroke and 36–48 h after stroke, 36–48 h and 3 months after stroke, 3 and 12 months after the stroke.

Dependency in ADL was evaluated for 253 patients with complete ADL data at four time points (Figure 3). Dependency was observed before stroke in 2% of patients and in 42, 13, and 11% of patients at 36–48 h, 3 months, and 12 months after stroke, respectively. The proportion of patients with ADL dependency was significantly increased at 36–48 h after admission (vs. before stroke, *p* < 0.001) and significantly lower at 3 months than at 36–48 h (*p* < 0.001) (Figure 3).

### Explaining ADL Dependency After 3 Months

Multicollinearity was not observed between the independent variables. The univariable model showed that ADL, as assessed with BI within 36–48 h after admission, was associated with significantly increased odds of dependency after 3 months (OR: 0.96, 95% CI: 0.94–0.97. AUC of the model 0.76), (Table 2). The multivariable model confirmed that ADL within 36–48 h after admission was associated with increased odds of dependency after 3 months (OR: 0.96, 95 % CI: 0.94–0.98. AUC of the model 0.80) (Table 2). Psychometric properties of the models as well as cross-validated models are presented in Table 2.

**Table 2:** Explaining dependency in activities of daily living 3 months after stroke.

	**B coefficient**	**OR (95% CI)**	***P*-value**	**Sensitivity, %**	**Specificity, %**	**Positive predictive value, %**	**Negative predictive value, %**	**Y_i_**	**AUC**
Univariable model				74.1(60.4–85.0)	64.1(57.9–69.9)	30.1(25.5–35.1)	92.2(88.2–94.9)	38.2	0.76(0.69–0.83)
ADL (BI, range 10–100p), per 5 gained points	−0.04	0.96(0.94–0.97)	<0.001						
Multivariable model				78.9(66.3–88.9)	72.4(66.5–77.8)	36.9(31.4–42.8)	94.4(90.8–96.6)	51.3	0.80(0.73–0.87)
ADL (BI, range 10–100p), per 5 gained points	−0.04	0.96(0.94–0.98)	<0.001						
Cognitive function (MoCA, range 3–30p), per 1 gained point	−0.10	0.91(0.85–0.97)	0.005						
Stroke severity (NIHSS, range 0–19 p), per 1 gained point	−0.03	0.97(0.87–1.07)	0.55						
Age (range 19–97), per 1 gained year	0.03	1.03(1.00–1.07)	0.03						
**10 – fold cross-validation (90% training set and 10% testing set), mean ± S.D**.									
				**Sensitivity**	**Specificity**	**Positive predictive value**	**Negative predictive value**	**Y**_**i**_	**AUC**
Univariable model			0.57 ± 0.10	0.81 ± 0.24	0.27 ± 0.07	0.94 ± 0.06	0.38	0.76 ± 0.08	
Multivariable Model			0.73 ± 0.07	0.70 ± 0.19	0.34 ± 0.07	0.92 ± 0.05	0.43	0.79 ± 0.11	

*Y_i_, Youden's index (sensitivity (%) + specificity (%) – 100[or 1 for cross validated results]); ADL, personal activities of daily living; BI, Barthel index, the score in BI is increased with 5 points; MoCA, the Montreal Cognitive Assessment; NIHSS, the national institutes of health stroke scale; BI and MoCA were administered 36–48 h after admission to the stroke unit. NIHSS, hospital admission point; BI and MoCA, a higher score indicates better outcome; NIHSS, a higher score indicates worse outcome; The results of tuned univariable and multivariable logistic regression models and 10-fold cross validation performed per respective model*.

### Explaining ADL Dependency After 12 Months

Multicollinearity was not observed between the independent variables. The univariable model showed that ADL as assessed with BI within 36–48 h after admission was associated with significantly increased odds of dependency after 12 months (OR: 0.95, 95 % CI: 0.94–0.97. AUC of the model 0.77), (Table 3). The multivariable model confirmed that ADL within 36–48 h after admission was associated with increased odds of dependency after 12 months (OR: 0.96, 95 % CI: 0.94–0.98. AUC of the model 0.80), (Table 3). Psychometric properties of the models as well as cross-validated models are presented in Table 3.

**Table 3:** Explaining dependency in personal activities of daily living 12 months after stroke.

	**B coefficient**	**OR (95% CI)**	***P*-value**	**Sensitivity, %**	**Specificity, %**	**Positive predictive value, %**	**Negative predictive value, %**	**Y_i_**	**AUC**
Univariable model				78.6(63.2–89.7)	63.3(56.8–69.4)	27.5(23.2–32.3)	94.3(90.3–96.8)	41.9	0.77(0.70–0.85)
ADL (BI, range 10–100p), per 5 gained points	−0.04	0.95(0.94–0.97)	<0.001						
Multivariable model				75.0(58.8–87.1)	71.9(65.6–77.6)	31.6(26.0–37.6)	94.3(90.6–96.6)	46.9	0.80(0.72–0.87)
ADL (BI, range 10–100p), per 5 gained points	−0.04	0.96(0.94–0.98)	<0.001						
Cognitive function (MoCA, range 3–30p), per 1 gained point	−0.06	0.94(0.87–1.01)	0.10						
Stroke severity (NIHSS, range 0–19 p), per 1 gained point	0.03	1.03(0.92–1.15)	0.62						
Age (range 19–97), per 1 gained year	0.04	1.04(1.01–1.08)	0.02						
**10 – fold cross-validation (90% training set and 10% testing set), mean ± S.D**.									
				**Sensitivity**	**Specificity**	**Positive predictive value**	**Negative predictive value**	**Y_i_**	**AUC**
Univariable model			0.57 ± 0.15	0.70 ± 0.40	0.22 ± 0.17	0.95 ± 0.06	0.27	0.76 ± 0.11	
Multivariable Model			0.70 ± 0.09	0.69 ± 0.32	0.29 ± 0.19	0.94 ± 0.06	0.39	0.80 ± 0.12	

*The results of tuned univariable and multivariable logistic regression models and 10-fold cross validation performed per respective model. Y_i_, Youden's index (sensitivity (%) + specificity (%) – 100[or 1 for cross validated results]); ADL, personal activities of daily living; BI, Barthel index, the score in BI is increased with 5 points; MoCA, the Montreal Cognitive Assessment; NIHSS, the national institutes of health stroke scale; BI and MoCA were administered 36–48 h after admission to the stroke unit. NIHSS, hospital admission point; BI and MoCA, a higher score indicates better outcome; NIHSS, a higher score indicates worse outcome*.

## Discussion

The results of this study indicated that dependency in ADL within 36–48 h after admission to the stroke unit could explain dependency in personal ADL at 3 and 12 months after stroke. These results are in line with previous findings that patients with ADL dependency after stroke were more likely to have long-term dependency (8, 31); nevertheless, these prior studies did not conduct very early ADL assessments after admission. A few studies have examined early ADL assessments for explaining long-term dependency; while their findings support our results, these studies evaluated patients with more severe strokes and greater ADL dependency at baseline (17, 32).

Performance and performance repertoire of activis of daily living is formed during the lifetime. When it comes to personal ADL, the performance becomes automatized during the lifetime, as activities are performed on daily basis from the very early age. This means that the cognitive and physical demanand are different. Due to automatization, the cognitive demand on personal ADL might be samwhat low, while physical demand still remains high.

We found that cognitive impairment was a significant explaining factor of ADL dependency 3 months after stroke, which is consistent with prior reports (9, 33). Cognitive impairment is a common sequalae after stroke and can persist even after seemingly successful neurological recovery. It can affect ADL performance, especially in the early stages after stroke. However, cognitive functions may exhibit long-term improvements (34), and patients may develop compensatory strategies for managing ADL. Moreover, in our study ADL comprised personal activities of daily living, such as mobility, using the toilet, and getting dressed and undressed. These tasks generally have low complexities as performance has been mastered during the lifetime (35). Hence, cognitive demand can be low. Taken altogether, these factors may explain the lack of a significant relationship between cognitive impairment and ADL dependency at 12 months after stroke.

The present study also revealed that older age significantly explained ADL dependency at 3 and 12 months after stroke; this is supported by the results of previous studies (8, 10). This relationship may be linked to typical age-related decreases in physical ability, which are thought to be associated with both aging and a higher number of comorbidities (36).

We found that stroke severity assessed with NIHSS did not explain ADL dependency at 3 or 12 months; this conflicts with previously reported results (10, 37). However, these studies had substantially higher median stroke severity values, while patients in our study had predominantly mild strokes (19). Thus, accurate explanation of ADL dependency in the later stages after stroke may not be possible if it is solely based on the NIHSS in a population in which most strokes are mild (19). Moreover, very early NIHSS might be an insignificant explanatory variable for stroke-related outcomes, as many people experience good neurological recovery after reperfusion treatment (38). In addition, the results of the present study indicated that while the proportion of patients with ADL dependency at 3 months was significantly lower than that at baseline, there was no significant difference between 3 and 12 months. This seems to support the belief that recovery primarily occurs early after stroke (13, 14).

The present study has several strengths and limitations. First, we did not have access to complete data regarding all variables, as we evaluated data from a national registry (22) and a research database (20). Second, the dependent variables (ADL dependency at 3 and 12 months after stroke) were based on self-reported data, and non-responders were excluded from the regression analyses. Although this exclusion may have resulted in selection bias, drop-out analyses revealed no significant differences in terms of baseline characteristics (e.g., age, sex, stroke severity, and BI score). Moreover, excluded patients did not differ from included patients in terms of age (according to the Swedish dementia registry, the median and mean age at first dementia diagnosis is 80 years). In addition, the severity of cognitive impairment, as well as stroke severity was not that high in the study sample. Therefore, there is a low probability that dropout could be explained due to dementia. The BI was used to assess ADL at baseline. While this tool is considered valid and reliable for evaluating stroke patients, it may have a ceiling effect in an acute care setting (39). In this study, four binary logistic regression models were built. Sensitivity, Specificity, PPV, and NPV were evaluated under cross-validation, where the threshold value was selected to optimize Y_i_. The number of variables in our logistic regression models were restricted by the fact that our sample had low proportions of ADL dependency at 3 and 12 months, similarly to the general population of Swedish stroke patients. A larger sample size might have permitted more variables to be used in the models; however, variable selection would be dependent on the algorithm of the binary regression analyses. As variable selection was based on a directed acyclic graph, which was based on clinical reasoning and prior studies, the results of the present study would be of high clinical relevance.

## Conclusion

The results of this study indicated that ADL dependency within the first 2 days after stroke may explain dependency in personal ADL 3 and 12 months later in a group of Swedish patients. These results, in addition to stroke severity and ADL at discharge, may help to increase understanding regarding rehabilitation needs and follow-up for patients with minor strokes, as their hospital stays have typically become shorter in recent decades. The external validation of the study results is recommended.

## Data Availability Statement

The datasets presented in this article are not readily available because according to the Swedish regulations (https://etikprovning.se/for-forskare/ansvar/), complete data cannot be made publicly available for ethical and legal reasons. However, researchers can submit requests for data to the principal investigator (contact: ks.sunnerhagen@neuro.gu.se). Requests to access the datasets should be directed to Katharina S. Sunnerhagen, ks.sunnerhagen@neuro.gu.se.

## Ethics Statement

The study was approved by the Gothenburg Regional Ethical Review Board (http://www.epn.se/sv/goeteborg/om-naemnden/, reference number: 042–11, amendment: T966-17). The Swedish Data Protection Authority does not require informed consent for research use of registry data. In addition, the Personal Data Act (Swedish law #1998:204, issued April 29, 1998) allows medical chart data to be collected for clinical research and quality control purposes without written informed consent. Written informed consent for participation was not required for this study in accordance with the national legislation and the institutional requirements.

## Author Contributions

HE: analysis and interpretation of the data and drafting of the manuscript. TA: acquisition of data, conceptualization of the study, analysis, interpretation of the data, and revising the manuscript for intellectual content. LR: acquisition of data, conceptualization of the study, and revising the manuscript for intellectual content. KS: design or conceptualization of the study, interpretation of the data, and revising the manuscript for intellectual content. All authors contributed to the article and approved the submitted version.

## Funding

This study was supported by grants from the Swedish Research Council (VR2017-00946), the Swedish Heart and Lung Foundation, the Swedish Brain Foundation, Promobilia, the Swedish state under an agreement between the Swedish government and the county councils, the ALF agreement (ALFGBG 71980, ALFGBG-877961), the Swedish National Stroke Association, the Local Research and Development Board for Gothenburg and Södra Bohuslän, the Center for Person-Centred Care (GPCC) at the University of Gothenburg, Greta and Einar Asker's Foundation, Rune and Greta Almöv's Foundation for Neurological Research, Hjalmar Svensson's Research Foundation, Herbert and Karin Jacobson's foundation, Doktor Felix Neubergh's foundation, and Gun and Bertil Stohne's foundation.

## Conflict of Interest

The authors declare that the research was conducted in the absence of any commercial or financial relationships that could be construed as a potential conflict of interest.

## Publisher's Note

All claims expressed in this article are solely those of the authors and do not necessarily represent those of their affiliated organizations, or those of the publisher, the editors and the reviewers. Any product that may be evaluated in this article, or claim that may be made by its manufacturer, is not guaranteed or endorsed by the publisher.
